# Surgeon-General Baxter

**Published:** 1891-01

**Authors:** 


Surgeon General Baxter, U. S. Army.—The ink on General Baxter's new Commission had scarcely dried, when the startling intelligence was sent out that he had been stricken with paralysis; and the attack proved fatal December 4th, 1890, with a return to partial consciousness only, and that of few moments' duration, from the inception to the ending of the disease.

Dr. Jedediah H. Baxter was a native of Vermont, and was fifty-three years of age at the time of his death. He was educated at the University of Vermont, where he took both the Academic and Medical degrees, and later the law degree of the Law School of Columbia University.

He was admitted to the bar of the Supreme Court, though his chosen profession was medicine, not law, and the breaking out of the war found him engaged in the practice of the former in the State of Massachusetts.

Dr. Baxter entered the military service in 1861, as Surgeon of Col. Fletcher Webster's 12th Massachusetts Regiment; in April, 1862, was appointed Surgeon United States Volunteers (Staff Surgeon); in 1864 was detailed for duty in the War Department as Chief Medical Officer of the Provost-Marshal General's Bureau; and in these several positions he acquitted himself with distinction. He saw service in the field with the Army of the Potomac, and had charge of a United States General Hospital in Washington, besides the experience of a Bureau Office in the War Department, all of which peculiarly well equipped him for his last and highest office. But he also held the posts of Assistant and Chief Medical Purveyor of United States Army, from the close of the war to the date of his promotion, August 16, 1890, where he further added to and developed his administrative talents. Under General Baxter's administration the Surgeon General's Department promised an able and progressive future.

His funeral was held from All Saints' Unitarian Church, December 6, 1890, and his remains found rest in that appropriate spot, Arlington Cemetery. The escort pertaining to his rank of Brigadier General, viz., five battalions of infantry, a platoon of artillery, and two troops of cavalry, served at the funeral, and the War Department closed at noon on the day thereof as a mark of respect.

